# Biodentine for Furcation Perforation Repair: An Animal Study with Histological, Radiographic and Micro-Computed Tomographic Assessment

**DOI:** 10.22037/iej.v13i3.19890

**Published:** 2018

**Authors:** Miguel Cardoso, Maria dos Anjos Pires, Vitor Correlo, Rui Reis, Manuel Paulo, Carlos Viegas

**Affiliations:** a *University of Trás-os-Montes e Alto Douro, School of Agrarian and Veterinary Sciences, Department of Veterinary Sciences, Quinta de Prados, Vila Real, Portugal; *; b * Health Sciences Institute of Universidade Católica Portuguesa, Department of Endodontics; Estrada da Circunvalação, Viseu, Portugal; *; c * 3B's Research Group-Biomaterials, Biodegradables and Biomimetics, Department of Polymer Engineering-School of Engineering, University of Minho, Guimarães, Portugal*

**Keywords:** Biodentine, Biomaterial, Endodontics, Furcation Perforation, Imaging, Micro-Computed Tomography

## Abstract

**Introduction:**

Biodentine has been scarcely studied as a furcation perforation (FP) repair material, mostly by *in vitro* methodologies. This animal study aimed to compare the histological responses, radiographic, and micro-computed tomographic (micro-CT) outcomes after FP repair with Biodentine or ProRoot MTA (MTA) in dogs’ teeth.

**Methods and Materials:**

Fifty teeth from five dogs were divided into 4 groups: MTA (*n*=20, FP repaired with ProRoot MTA), BDT (*n*=20, FP repaired with Biodentine), PC (*n*=5, positive control, FP without repair) and NC (*n*=5, negative control, without perforation). The animals were euthanized after 4 months. Histological assessment included inflammatory cell infiltration, hard tissue resorption, hard tissue repair, and cement repair in the furcation area. Immediate postoperative and 4 months follow-up radiographs were compared for radiolucency in the furcation region. The volume of extruded material was quantified using micro-CT images.

**Results:**

The tested materials showed equivalent radiographic response, together with similar hard tissue resorption and repair but, BDT group showed significantly less inflammation, lower volume of extruded material and higher cement repair than MTA group.

**Conclusion:**

The outcomes of this study, taken together with other favorable results in literature, are highly suggestive that Biodentine is a promising biomaterial to be used for FP repair.

## Introduction

Furcation perforations (FP) are anomalous communications between the root canal system and the external dental surface in the inter radicular region of multi-rooted teeth, connecting the pulp cavity with periodontal tissues [1, 2]. 

Current advances in endodontics and biomaterials made the recovery of tooth structure and function possible even in the most complicated cases. Despite the breakthroughs in techniques and resources, FP management remains challenging [3, 4] and the best approach is still unclear. Unintentional extrusion of the repair material, inadequate sealing and lack of biocompatibility are among the described difficulties [5, 6]. Available options are mostly based on inconsistent clinical reports, raising the need for *in vitro* and *in vivo* studies under controlled conditions that simulate the clinical features of this pathology.

Two main techniques have been advocated for the repair of such defects. Since surgical procedures for FP repair may induce pocket formation, nonsurgical methods-especially in inaccessible areas- are favored [3, 7]. Ideally, perforations should be immediately repaired with a biocompatible material to seal the communication between the perforation site and the gingival sulcus in order to achieve a more favorable prognosis [3, 8].

Mineral trioxide aggregate (MTA) is a bioactive material which became available for dental practice in 1993. MTA powder is mainly a mixture of tricalcium silicate, tricalcium aluminate, tricalcium oxide and silicate oxide, with bismuth oxide added as a radio-opacifier agent. Because of their biocompatibility and bioactivity, MTA formulations, including ProRoot MTA (MTA) (Dentsply, Tulsa/Dental, Tulsa, OK, USA), have become the most common choice for FP repair [9]. 

Biodentine (Septodont, Saint-Maur-des-Fosses, France) is a bioactive cement, in which the main component of the powder is tricalcium silicate, with addition of zirconium dioxide and calcium carbonate; the liquid has calcium chloride as a setting accelerator. Biodentine has been reported to provide good biocompatibility, bioactivity [10, 11], high compressive strength and a short setting time of 12 min [12, 13]. Since its approval for dental use by the FDA in 2009, Biodentine was widely studied for several applications, however its use in FP has been scarcely addressed, mostly by *in vitro* studies [4, 14-22]. Despite the promising results in this field, further studies are necessary with larger *in vivo* samples, stronger methodologies and complementary assessment, including imaging. 

The aim of this animal study was to compare the histological responses, radiographic, and micro-computed tomographic (micro-CT) outcomes after FP repair with Biodentine or ProRoot MTA in dogs’ teeth. In this context, the null hypothesis stated that there would be no significant differences in the histological or imaging findings of Biodentine or MTA. 

## Materials and Methods

All animal procedures were approved by the institutional Ethical Committee (Edifício Pedrinhas, Universidade de Trás-os-Montes e Alto Douro, 5001-801 Vila Real, Portugal) and followed the ethical guidelines and regulations of the national Directorate-General for Food and Veterinary (Process number 0421/000/000/2014), and with the ones laid down by the European Union Directive 2010/63/EU for animal experiments. All measures were undertaken to minimize pain and animal discomfort.

The second and third maxillary premolars and the second, third, and fourth mandibular permanent premolars of 5 male beagle dogs, aging 18 months and weighing 17 kg on average, were selected for the study, providing a total of 50 teeth. 

The teeth were divided into two experimental groups, MTA (FP repaired with ProRoot MTA) and BDT (FP repaired with Biodentine), with 20 teeth each, and two control groups, PC (positive control, with FP without repair) and NC (negative control, without perforation), with 5 teeth each. Each animal had 4 teeth repaired with Biodentine, 4 with MTA, 1 positive control and 1 negative control, randomly assigned within the teeth included in the study. Table 1 summarizes the experimental groups and the 50 teeth distribution.

All procedures were carried out under general anesthesia. Each animal was pretreated with 0.2 mg/Kg morphine, 0.005 mg/Kg acepromazine and 0.004 mg/Kg dexmedetomidine, administered intramuscularly, and general anesthesia was induced with intravenous 0.5 mg/Kg ketamine, 0.2 mg/Kg diazepam and 2 mg/Kg propofol. The animals were intubated with a cuffed endotracheal tube, and anesthesia was maintained with isoflurane and a constant rate infusion of 10 μg/Kg/min of ketamine. Adequate prophylactic antibiotic and analgesia were provided. 

Preoperative standardized periapical radiographs were performed using film-holding devices. After prophylaxis and root scaling, all teeth were isolated with a rubber dam and the operative field was disinfected with 5% iodine. 

**Table 1:** Distribution of the 50 teeth included in the study by the experimental groups

**Experimental groups**	**Procedures**	**N per dog** †	**Total N per group**
**BDT **	FP repaired with Biodentine	4 teeth‡	20
**MTA**	FP repaired with ProRoot MTA	4 teeth‡	20
**PC**	Positive control with FP without repair	1 tooth‡	5
**NC**	Negative control without perforation	1 tooth‡	5

*n: number; *

†: *number of teeth in each of the 5 dogs used in this study; BDT: Biodentine group; FP: Furcation perforation;*

‡: *randomly assigned within the teeth included in the study for each dog; MTA: ProRoot MTA group; PC: Positive control group; NC: Negative control group*

**Table 2:** Histological assessment results

**Histology**	**BDT (** ***n*** **=18)**	**MTA (** ***n*** **=17)**	**PC (** ***n*** **=5)**	**NC (** ***n*** **=5)**
**Inflammation scores 1/2/3/4 (n)**	16/2/0/0	11/3/3/0	0/0/1/4	5/0/0/0
**Hard tissue resorption Yes/No (n)**	1/17	4/13	5/0	0/5
**Hard tissue repair Yes/No (n)**	18/0	17/0	0/5	NA
**Cementum repair at furcation area Scores 1/2/3/4 (n)**	9/8/1/0*	4/7/6/0*	0/0/0/5	NA

*BDT: Biodentine group; n: number; MTA: proRoot MTA group; PC: Positive control group; NC: Negative control group; NA: not applicable. *

* *Significant difference between test materials (P<0.001)*

**Figure 1:**
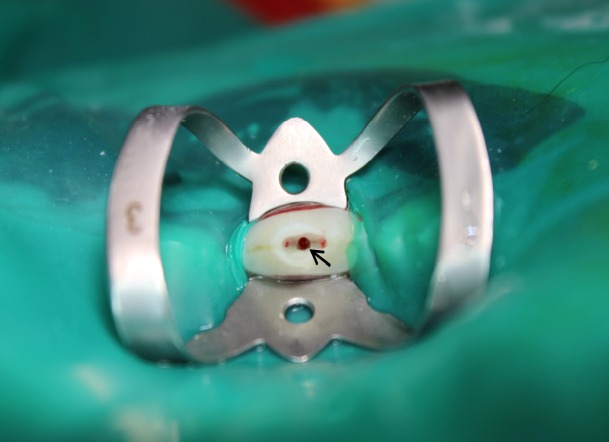
Access cavity with furcation perforation (arrow)

In the test and PC groups, access cavities were prepared in occlusal surfaces using a round diamond bur (ISO 012) in a high-speed handpiece under copious water spray. After complete removal of the pulp chamber roof, coronal pulp tissue was extirpated using a #10 curette up to the canal entrance level. A 1.2 mm diameter perforation was created in the center of the pulp chamber floor, using a sterile round bur (ISO 012) at low speed until hemorrhage was noted (Figure 1). Hemostasis was achieved with abundant saline irrigation and sterile cotton pellets.

Both cements (Biodentine and MTA) were prepared according to the manufacturers’ protocol, placed into the perforation defects and over the remaining radicular pulp tissue of the respective test group’s teeth and gently compacted with a plugger. After the initial setting of the materials, the access cavity was sealed with light-cured glass ionomer cement (Riva Light Cure LC/Southern Dental Industries SDI) and the teeth were radiographed again.

The PC perforation was left open. The NC group teeth received no intervention, to evaluate the potential effects of external variables that could have developed during the study period.

After 120 days, new periapical radiographs were performed and the animals were euthanized by sodium pentobarbital overdose. Each experimental tooth with the surrounding tissues was cut in a block section and placed in 10% buffered formalin.

Fixed block sections were scanned using a high-resolution micro-CT system (vivaCT 80, ScancoMedical) with the root oriented vertically. The x-ray transmission was set at 90 degrees rotation, with the x-ray source set at 70 kVp/114 μA, 8W. The scanning time for each sample was approximately 35 min.

The specimens were demineralized using Morse’s solution (50% formic acid and 20% sodium citrate) for three months. After complete decalcification, the specimens were dehydrated, embedded in paraffin and serially sectioned (3 m thickness). All sections passing through the FP site were stained with hematoxylin-eosin. Samples of these sections were stained with gram staining for histo-microbiological analysis.

***Radiographic assessment***

The immediate postoperative radiographs and those after 4 months were analyzed to determine the development or increase of radiolucency in the furcation region bordering the repaired perforations. 

This radiographic evaluation was performed blindly by two independent experienced dentists. For cases where evaluation did not match, were discussed to reach a consensus. 

***Micro-CT***

In the test groups, the volume of material that extruded to the periodontal tissues area was quantified for each tooth using micro-CT images.

***Histological assessment***

The specimens were blindly evaluated by two experienced oral pathologists under an optical microscope. The cases which evaluation did not match were discussed to reach a consensus. 

The connective tissue reactions and the periodontal specific reparative tissue response were evaluated under microscopy according to established criteria for inflammatory cell infiltration, hard tissue resorption, hard tissue repair and cementum repair in the furcation area.

Grading the parameter *inflammatory cell infiltration* was performed according to Ørstavik and Mjör as described by Noetzel [3]: *grade 1* - no detectable cells, *grade 2* - mild (few scattered inflammatory cells), *grade 3* - moderate (focal accumulation of inflammatory cells) and *grade 4* - severe (dense infiltration of inflammatory cells). 

The analysis of hard tissue response was performed according to Zairi* et al. *[23]: *Hard Tissue Resorption,* The changes were classified as “yes” or “no” based on whether there was tissue resorption in both bone and cementum adjacent to the amputated area. *Hard tissue repair,* the changes were classified as “yes” or “no” based on the presence or absence of tissue repair in both bone and cementum adjacent to the amputated area.

Grading the parameter “*Cement repair at furcation area*” was performed according to the score: *grade* 1 - totally repaired, *grade *2 - repair up to half of furcation, *grade* 3 - repair up to a quarter of furcation and *grade* 4 - no repair.

***Statistics***

Statistical analysis was performed using SPSS (version 22, SPSS, Inc., Chicago, IL, USA). Generalized Estimating Equations approach was used to analyze data. All differences were considered significant at *P*≤0.05. 

Values are expressed as means±standard deviation, median and interquartile range (IQR) or number (percentage), as appropriate.

**Figure 2:**
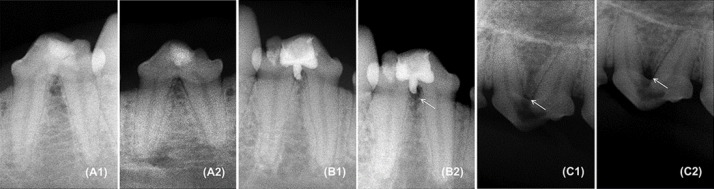
Radiographic images. *A)* BDT group specimen: *A1)* immediate postoperative, *A2)* 120 days after furcation perforation repair; *B)* MTA group specimen: *B1)* immediate postoperative, *B2)* 120 days after furcation perforation repair with development of radiolucency (arrow); *C)* PC group specimen: *C1)* immediate postoperative with radiolucency (arrow), *C2)* 120 days after furcation perforation with increase of radiolucency (arrow

## Results

***Clinical and macroscopic observations***

The animals remained healthy throughout the study period. No signs of infection or of local intolerance were observed. No deaths occurred. As expected, all the animals awoke uneventfully from anesthesia and reached the defined end-point (4^th^ postoperative month).

***Radiographic assessment***

Six MTA specimens (30%) presented development or increase of radiolucency in the furcation area, in contrast with only two cases (10%) in BDT group. Both test materials showed similar radiographic evolution 4 months after surgery (*P*>0.05) (Figure 2).

***Micro-CT***

**Figure 3:**
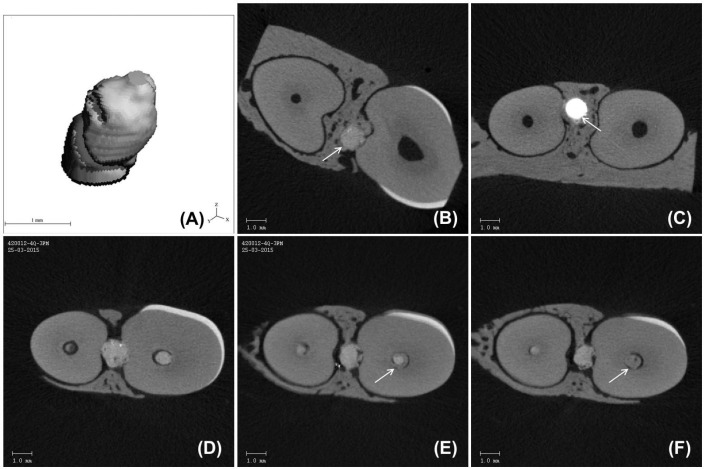
Micro-CT volume reconstruction and axial sections. *A)* Micro-CT 3D model reconstruction representative of extruded material volume; *B)* Micro-CT axial section of a tooth restored with Biodentine (arrow) in continuity with adjacent bone; *C)* Micro-CT axial section of a tooth restored with MTA (arrow) in continuity with adjacent bone; *D, E, F)* Micro-CT axial sections of a tooth restored with Biodentine showing dentine bridge (arrow) formation from coronal to apical (from D to F

Total volume of extruded material (Figure 3A) was significantly lower in BDT group than in MTA group (BDT: 1.42±0.80 mm^3^; MTA: 2.27±1.67 mm^3^; *P*=0.02). 

In both test material groups, micro-CT showed continuity between the extruded repair material and the surrounding bone (Figure 3B and C). Along with the study’s included outcomes, further evaluation of micro-CT images allowed the identification of new mineralized tissue bridges over the remaining radicular pulp tissue in specimens of both test groups (Figure 3D to F).

***Histological assessment***

**Figure 4:**
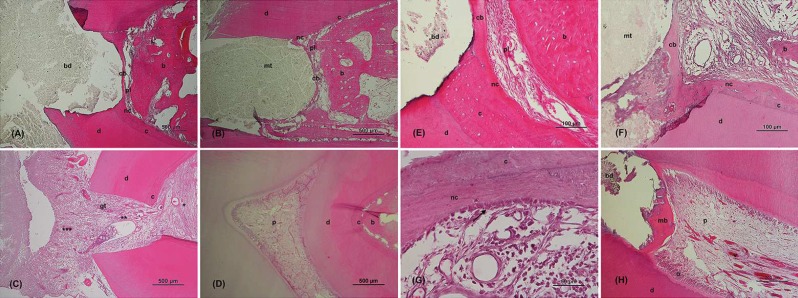
Histological images 120 days after furcation perforation repair. *A, E)* BDT group specimens in different magnifications; *B, F, G)* MTA group specimens in different magnifications; *C)* PC group specimen; *D)* NC group specimen; *H)* Mineralized bridge over vital pulp in a BDT group specimen. Conventional light microscopy; Hematoxylin-eosin; *A, B, C, D)* ×4 magnification; *E, F)* ×20 magnification; *G)* ×40 magnification; *H)* ×10 magnification. (Arrow: cementoblasts; *: furcation; **: perforation; ***: pulp chamber; b: bone; *bd*: Biodentine; *cb*: cementum bridge; c: cementum; *d*: dentin; *gl*: granulation tissue; *mb*: mineralized bridge; *mt*: MTA; *nc*: new cementum; o: odontoblasts; *p*: vital pulp; *pl*: periodontal ligament; *v*: blood vessels

From the 50 teeth, 3 specimens from MTA group and 2 specimens from BDT group, were excluded from the histological assessment due to technical problems during processing. Table 2 summarizes the results of the variables of histological assessment and Figure 4 shows illustrative histological images. As for micro-CT, further histological observation also identified new mineralized tissue bridges over the remaining radicular pulp tissue in specimens of both test groups (Figure 4H). There was no evidence of bacterial presence in test groups. 

***Inflammation scores***

Concerning the grade of inflammation 4 months after FP repair, both test groups presented favorable results with only two cases of few scattered inflammatory cells in BDT group and six cases in MTA group. Repair with Biodentine led to significantly lower inflammation scores than MTA (*P*=0.015), with median (IQR) score 1 (0) for BDT group and 1 (1) for MTA group. Median score for the PC group was 4 (1) and all NC samples were graded 1. Both test groups showed significant difference when compared to the positive control (BDT *vs.* PC: *P*<0.001; MTA *vs.* PC: *P*=0.001). Regarding the negative control group, MTA group showed significantly higher inflammation scores (*P*=0.016) while no statistically significant difference was found for BDT group (both *P*>0.05).

***Hard tissue resorption***

After 4 months, resorption was present in all the specimens of the PC group (Figure 4C), in none of the NC group (Figure 4D), in four MTA group specimens and in only one case in the BDT group, however without significant difference (*P*>0.05) between test-material groups.

***Hard tissue repair***

At the fourth postoperative month, all specimens of the MTA and BDT groups showed signs of hard tissue repair (Figure 4G). 

***Cementum repair at furcation area***

Four months after FP repair, all the test groups’ specimens presented cementum formation (Figure 4A, B, E and F). BDT group showed significantly better scores than MTA group [Median scores (IQR); BDT: 1.5 (1)* vs.* MTA: 2 (2), *P*=0.04]. The two groups presented significantly higher repair than the PC group (BDT *vs*. PC: *P*<0.001; MTA *vs*. PC: *P*=0.005). As expected, all PC samples were graded 4, with no cementum repair.

## Discussion

This report presents an *in vivo* qualitative and quantitative analysis of histological and imaging findings after FP repair with Biodentine, using an established large animal model. In this study, Biodentine behavior was at least comparable to the gold standard, and eventually better. 

Different materials have been used for perforation repair and their performance has been assessed through several methodologies; however, so far, none was considered as the ideal [2, 3, 7, 8, 19]. In cases where there is direct contact with the surrounding connective tissue, biocompatibility is of primary significance.

In this study, MTA was used as a comparison material; because, despite drawbacks such as poor handling characteristics and slow setting time [9, 20], it has been considered a gold standard in FP repair [9], due to the favorable sealing abilities and high biocompatibility [2, 3, 8, 9]. 

Even though Biodentine did not reveal favorable results in washout tests in one study [24], various other tests published in literature revealed promising results, such as increased compressive and push-out bond strength [12, 20], high density, decreased porosity [25], and microleakage [26], color stability [27], induction of cell proliferation and biomineralization [11, 25, 28-30], immediate formation of calcium hydroxide, high release and depth of incorporation of calcium ions [31], low cytotoxicity [10, 32], gingival fibroblast viability preservation [29], ease of handling and faster setting time (12 min) [13, 24, 31], and wide clinical applicability [12, 13, 33, 34]. Hence, Biodentine could be an efficient alternative to mineral trioxide aggregate formulations [13].

The favorable results regarding inflammation scores and hard tissue analysis support biocompatibility and bioactivity of both materials in FP repair, which is in consonance with previous general studies regarding Biodentine [10-13, 22] using different methodologies. Also, even though it was not part of study’s outcomes, the observation of new mineralized tissue bridges over the remaining radicular pulp in the test groups is consistent with other findings in literature, namely an animal study by Rossi *et al.* [31], who used Biodentine and MTA after pulpotomy in dogs and described similar results, demonstrating tissue compatibility. 

Concerning radiographic assessment results, when the furcation perforations were performed, a small defect was occasionally created in the bone, viewable on the postoperative radiographs. The absence of significant difference in radiological examination between the test groups was consistent with the histological results.

Micro-CT is a non-destructive technique that does not require specimen demineralization and therefore can provide additional information [35]. 

In this study, micro-CT was used to quantify the volume of extruded material to the periodontal tissues. Extrusion of filling material into the periodontal space may hinder periodontal reattachment [36] and adversely affect tissue repair [8], since there is no absorption of the material by the tissues [37]. This is even more relevant for FP which, given their anatomical specificities, act as bottomless pits [38]. The use of internal biocompatible matrices has been advocated to help control the extrusion of the filling materials as well as increase their sealing ability, however with equivocal results [6, 39]. The greater amount of extruded material found for MTA group is consistent with its lengthier setting time, which may contribute to the unintended compaction of the unset material into the furcation defect. 

The higher grade of cementum repair found in BDT group may be explained by lesser amount of extruded material than in MTA group. Also, the difference in the speed of chemical reactions occurring during the setting of materials is considered to be associated with better repair conditions. Although both materials produce the same chemical compounds [25], Biodentine allows a greater release of ions during the initial setting, which reduces over time [40], subsequently improving conditions [13]. Moreover, hydration of the calcium oxide contained in MTA may prompt an exothermic reaction [25], possibly inducing less favorable conditions [31]. It may be hypothesized that if the study had a longer evaluation period, the overall results for MTA and BDT groups could be even more similar.

Regarding the hard tissue repair found in all test group specimens, although the perforations were done using slow speed, temperature may have increased when inserting the bur into the alveolar bone, generating low thermal trauma [2, 6, 7], with subsequent resorption and deposition of new bone and cementum, as hypothesized by Al-Daafas *et al.* [6]. 

According to the results of this study, both MTA and Biodentine yield acceptable results in FP repair in dogs’ teeth. Dogs were selected for this study because of their well-documented physiological responses and dental anatomy, with a suitable furcation that provides good accessibility and visibility. However, the furcation of the posterior teeth is often as close as 1 to 2 mm from the cemento-enamel junction [6]. Therefore, epithelialization of a furcation perforation in dogs is considered more likely than in human teeth, where the furcation is deeper in the alveolus. Thus, our favorable results obtained in a dog model of FP may be associated with even better responses in humans [6]. Nevertheless, extrapolation of our data to a clinical setting must be cautious, as with every animal study.

## Conclusion

It may be concluded that Biodentine presented tissue compatibility and allowed for mineralized tissue formation after FP repair in dogs’ teeth, with similar morphology and integrity but greater cement formation than MTA. The excellent outcomes of the present study, complementing other favorable results obtained by different research methods, are highly suggestive that Biodentine is a promising biomaterial to be used for the repair of furcation perforations.
